# I’m No Superman: fostering physician resilience through guided group discussion of *Scrubs*

**DOI:** 10.1186/s12909-021-02856-9

**Published:** 2021-08-06

**Authors:** Arthur Holtzclaw, Jack Ellis, Christopher Colombo

**Affiliations:** 1 Department of Medicine, Pulmonary/Critical Care, Walter Reed National Military Medical Center, Uniformed Services University of Health Sciences, 8901 Rockville Pike, Bethesda, MD 20889 USA; 2 Hospitalist Division, Department of Medicine, Medical College of Georgia Augusta University, Augusta, GA USA; 3 Department of Medicine, Virtual Health and Tele Critical Care, Madigan Army Medical Center, Uniformed Services University of Health Sciences, Bethesda, MD USA

**Keywords:** Physician wellbeing, Burnout, GME, Curriculum, Media in education

## Abstract

**Background:**

Almost half of trainees experience burnout during their career. Despite the Accreditation Council on Graduate Medical Education (ACGME) recommendation that training programs enact well-being curricula, there is no proven method of addressing this difficult topic.

**Methods:**

We created a curriculum addressing physician resiliency and well-being, designed for an Internal Medicine Residency Program. This curriculum utilized episodes from a medical television series, *Scrubs*, to facilitate a monthly, 1-h faculty guided discussion group. We collected informal feedback and abbreviated Maslach Burnout Inventories (aMBI) monthly and conducted a formal focus group after 6 months to gauge its effectiveness.

**Results:**

The curriculum was successfully conducted for 12 months with each session averaging 18–20 residents. Residents reported high satisfaction, stating it was more enjoyable and helpful than traditional resiliency training. 19 of 24 residents (79 %) completed a baseline aMBI, and 17 of 20 residents (85 %) who attended the most recent session completed the 6-month follow-up, showing a non-significant 1-point improvement in all subsets of the aMBI.

**Conclusions:**

This novel, low-cost, easily implemented curriculum addressed resiliency and burn-out in an Internal Medicine Residency. It was extremely well received and can easily be expanded to other training programs or to providers outside of training.

**Supplementary Information:**

The online version contains supplementary material available at 10.1186/s12909-021-02856-9.

## Background

Physician burn-out (PBO) may be generally described as a state of emotional, physical, and mental exhaustion caused by excessive and prolonged stress [1]. PBO is directly linked to decreased job satisfaction, increased preventable errors, worse patient outcomes, and early retirement [2]. Unfortunately, it affects physicians at all levels with a prevalence of approximately 45.2 % among trainees [3]. Because of this, the Accreditation Council on Graduate Medical Education (ACGME) has made trainee well-being a priority with recommendations for training programs to develop strategies addressing the issue.

Programs designed to mitigate PBO commonly include self- or group-reflection [4]. However, due to a lack of symptom recognition, or fear of appearing weak there is often reluctance, especially among trainees, to acknowledge PBO and mental health issues [5, 6]. Dyrbye et al. found that only 27 % of medical students would definitely seek help for a severe emotional problem compared to 44 % of the general population [5]. In a second study, Schwenk et al. report medical students being unlikely to seek help due to perceived stigma and fear of adverse effects on their career [6]. Unfortunately, 56 % of medical students also reported witnessing other students reveal the mental health challenges of their peers and that making it less likely for them to seek help [5]. Thus, a major challenge for well-being programs is creating an environment of trust and safety among physicians to encourage participation while a key goal is to encourage open conversation and lessen the stigma of PBO/mental health issues.

On review of the literature, we noted several examples of using media to teach aspects of medicine, most commonly professionalism and communication skills, though we found no examples of media being used for PBO [7, 8]. Of note, few of these studies measured the outcomes they sought to improve, but rather the enjoyment of the experience as an alternative to a more traditional curriculum [9, 10]. These programs typically used 1–2 min illustrative clips with a reflection or discussion component, while more rarely, a full length movie might be used to illustrate a longer story that still had topical relevance [11]. However, no longitudinal character studies were found.

Given the documented learner satisfaction with the use of popular media, we elected to create a longitudinal monthly curriculum combining the viewing of selected media with group discussions related to topics on PBO. The primary goal of our investigation was to determine the feasibility of a regular facilitated discussion session utilizing themes and events in a fictional comedic medical television program in our internal medicine training program. Our secondary goal was (if the intervention demonstrated feasibility) to elicit and record the qualitative response to the intervention in our trainees. Thirdly we sought to measure the quantitative impact of the intervention on the self-report of burnout using a standardized reporting instrument.,

## Methods

Based on lessons learned from these prior studies, we established a conceptual basis for this pilot with a set of characteristics that would be required of the curriculum: (1) media reflecting a sincere, honest and relatable portrayal of the struggles faced by physicians in training (2) longitudinal character development throughout the spectrum of training levels. (3) a show that is entertaining and enjoyable (4) a process to specifically measure PBO as well as subjective experience of the trainees. We chose a television program to allow for brief, episodic sessions that could longitudinally follow recurring character portrayals and growth. The multiple seasons of the show allowed for a developmental view reflecting longitudinal professional and emotional development of our trainees. This was felt to prevent the perception of the curriculum as being for a particular year and to engage the multiple years represented in our mixed audience by relating to the experiences of the characters either in the observer’s past, present or future.

**Fig. 1:**
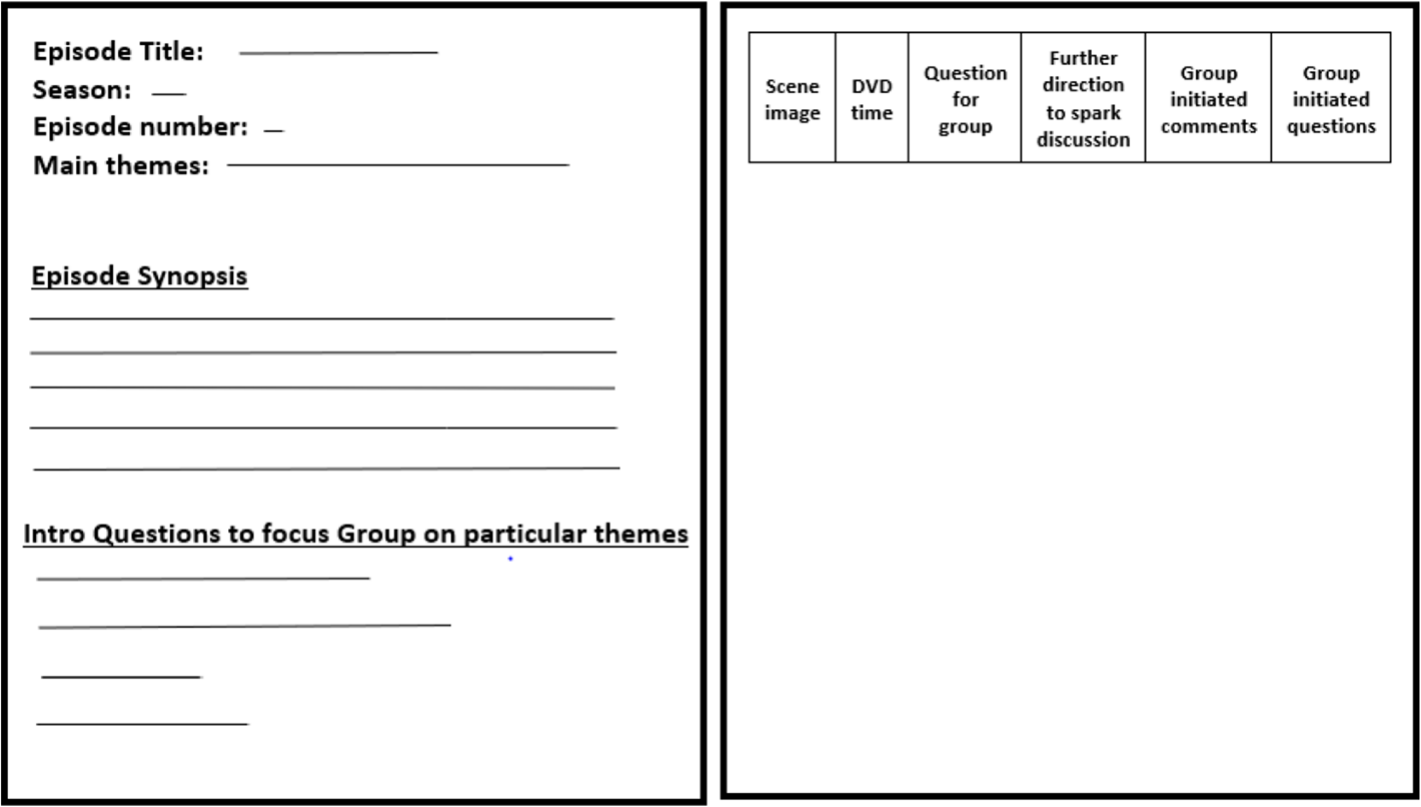
Sample episode guide with the front giving an overview of the episode with introduction questions and the back giving exact stop times and specific questions to help guide the discussion. See Additional file 1 for our full episode guides

*Scrubs* is a popular medical drama anecdotally felt to most accurately depict the stress and emotions common within a Medicine residency. As such, we pre-selected certain episodes to address topics associated with PBO such as physician cynicism, dealing with death, and work-life balance. The episodes last on average 23 min, allowing for 37 min for discussion during and after the episode. An episode guide (Fig. 1 and Additional file 1) was developed for each monthly session that describes the session goals, provides an episode introduction, and designates pre-set stopping points with open-ended questions to help the faculty facilitate conversation. However, the residents were encouraged to stop the episode themselves if they had something to discuss and the facilitators had complete flexibility in how they conducted the session based on the flow of conversation. The guides covered a multitude of issues within the episode, and when feasible, they were organized in two parallel formats: the complete 23-min episode, or a focused sub-plot guide. The sub plot guide has start and stop times for select DVD scenes to allow focus on a particular topic or conversely compress the presentation and allow more discussion time.

The sessions were conducted in the residency conference room during lunch prior to an already protected academic half day. To mitigate the additional hour of required time, food was provided for each session and the inpatient pagers were held by the team attending physicians. The protected time is essential as it allowed the residents to relax and prevented frequent disruptions of the discussion. Logistical requirements include a room big enough to fit the group, a DVD player and projector/screen to view the episode, and the *Scrubs* DVD for the episode.

Qualitative feedback was obtained in three different manners. First, the session leader asked directly after the sessions for any feedback on their conduct, perceived utility, and trainee reactions. The session leader also emphasized resources available for any trainee struggling with PBO or mental health issues. Second the program director utilized an end of week residency sync session following morning report to ask for feedback about the session during the weeks they occurred, which allowed some time for the trainees to reflect. Third, after 6 sessions were complete, the Chief Resident utilized part of a monthly house-staff meeting to conduct a focus group to solicit detailed feedback and assess the longer-term curriculum impact. This meeting consisted of 11 trainees and consisted of the following questions with accompanied informal discussion: what is your opinion of the new curriculum? how does it compare to prior curricula on PBO that you have experienced? does it have a positive or negative effect on your work day? what are the strengths/weaknesses of the curriculum? do you feel it has improved your awareness of PBO in yourself/your peers? do you think we should continue this curriculum?

Additionally, online abbreviated Maslach Burnout Inventory (aMBI) were sent to the residents monthly. The aMBI is a 9-item questionnaire with 3 questions addressing each of 3 subsets, Emotional Exhaustion (EE), Depersonalization (DP) and Personal Accomplishment (PA) [12]. Questions are scored from 0 to 6 totaling 0–18 for each subset (Table 1). EE and DP scales are expected to increase with increasing burnout; PA scores should decrease with increasing burnout. The aMBI has been validated against the full Maslach Burnout Inventory [13] and is frequently used in GME literature due to its ease of use. The cutoffs for burnout vary widely in the literature, with three recent studies using a cutoff for DP of 7, 10 and 13 respectively [14–16].

**Table 1:** The Abbreviated Maslach Burnout Inventory. Each question is scored within a specific subset - Personal Accomplishment (PA), EE (Emotional Exhaustion), DP (Depersonalization)

	How often:	Never	A few times per month	Once a month or less	A few times a month	Once a week	A few times a week	Every day
	Category	0	1	2	3	4	5	6
I deal very effectively with the problems of my patients	PA							
I feel I treat some patients as if they were impersonal objects	DP							
I feel emotionally drained from my work	EE							
I feel fatigued when I get up in the morning and have to face another day on the job	EE							
I’ve become more callous towards people since I took this job	DP							
I feel I’m positively influencing other people’s lives through my work	PA							
Working with people all day is really a strain for me	EE							
I don’t really care what happens to some patients	DP							
I feel exhilarated after working closely with my patients	PA							

IRB review at Eisenhower Army Medical Center approved all methods and deemed the curriculum to be quality improvement and not human subjects research. Written informed consent was waived by the Eisenhower Army Medical Center IRB given the quality improvement determination and as all surveys were fully anonymous and optional. All methods were carried out in accordance with relevant guidelines and regulations.

## Results

We successfully implemented the curriculum on a monthly basis for 12 months in 2017. The sessions averaged 18–20 out of 24 Internal Medicine trainees (approx. 75–85 % of the training program) and became one of our most popular and highest attended conferences.

Feedback from the trainees was overall positive, stating the sessions “provide a unique forum to discuss common issues” and “generate good discussion among residents on topics that may otherwise go unrecognized.” One trainee cautioned sessions “allow residents to get emotional which can be productive but also disruptive to the day” while another described the sessions as “just another required event”. However, the trainees unanimously chose to continue the sessions, finding them overall “helpful” and better than other attempts at addressing PBO. Specifically, they reported that the lighthearted nature of the show and the informal nature of the sessions made it easier to discuss difficult topics than in a traditional didactic lecture. They also reported that having multiple levels of trainees was beneficial as it helped show that they were not alone in having these emotions and challenges. Junior trainees reported feeling validated when sharing an experience and hearing senior residents reporting similar experiences, helping normalize the discussion of PBO. Senior residents felt valued as mentors and reported honing leadership and team-building skills. Several residents also reported that this curriculum sparked conversations regarding PBO outside of the facilitated discussions, which was a key curriculum goal. In fact, one of the main topics the trainees elected to talk about at their trainee-only annual retreat was PBO and its effect on their personal and professional lives.

Nineteen of twenty-four residents (79 %) filled out a baseline aMBI in December and 17 of 20 residents (85 %) who had attended a session in the prior 4 weeks filled it out in June. No aMBI were obtained after June. The June aMBI showed a non-significant improvement of at least a one-point in each subscale compared to December (Table 2). To protect anonymity, no attempt was made to correlate session attendance with individual aMBI changes.

**Table 2:** Average scores for each subset of the aMBI at the beginning and six months into the curriculum. The three subsets are Personal Accomplishment (PA), Emotional Exhaustion (EE), and Depersonalization (DP)

	December	June	Absolute change	% Change
Total Residents	19	17		
Average PA	12.16	14.2	2.04	16.8 %
Average EE	10.95	9.94	-1.01	-9.2 %
Average DP	7.37	5.94	-1.43	-19.4 %

Several key lessons emerged. First, the most profound discussions were often sparked by the preceptor simply stopping the episode after a key moment and utilizing uncomfortable silence. Second, as above, having trainees from different year groups was beneficial as it allowed them to give personal accounts of how they overcame challenges, allowing for a more natural discussion of different resilience strategies than didactic approaches. Lastly, the longitudinal nature of the program allowed discussion of not only the acute stressors that our trainees encounter (unexpected death, poor interactions with families, etc.) but also the grinding impact that repeated “insults” can have on well-being as is readily evident on the show.

## Discussion

To our knowledge, this longitudinal utilization of a humorous television program with a guided open discussion about resiliency and provider well-being is the first of its kind. It was not only well-liked but also anecdotally successful in sparking candid communication about difficult topics both during the sessions and afterwards as well.

These observational results correlated well with the non-significant improvement in the aMBI scores after 6 months of the curriculum, most notably the improvement of each subscale by > 1 point. While there is no gold-standard for “clinically significant” changes in aMBI score, it can likely be extrapolated from the larger Maslach Burnout Inventory (MBI). Investigators have reported correlation between MBI scores and medical errors in Internal Medicine residents, showing an 9 % increased risk for medical errors for each 1 point increase in the DP subset and a 6 % increased risk for each increase in the EE subset [17]. This indicates that a 1 point difference as seen in this pilot study could be considered clinically relevant.

This was a pilot project with the typical associated limitations. The objective data from the aMBI did not reach statistical significance due to the small number of residents. Although the single site and specialty may limit generalizability we submit that PBO is so prevalent among medical personnel that this curriculum could be utilized by any specialty. Other confounders include possible seasonal variability in burnout (e.g. holiday blues, or pre graduation euphoria). We also acknowledge the possibility that the residents’ enjoyment and eagerness to continue could result from a desire to watch TV at work rather than any true improvement in resiliency.

However, this pilot demonstrated the feasibility of this curriculum and has laid the groundwork for widespread implementation and more rigorous study. This curriculum is easy to implement and popular among residents with the only cost being 1 h per month and the cost of the Scrubs DVD. The ready-made episode guide concept allows for multiple faculty perspectives to be utilized in the teaching sessions, is editable and customizable for specific program needs, and allows multiple focus areas for physician resilience. The subject matter itself is widely varied in topic and includes end of life care, dealing with death, diversity issues, as well as physician burnout and resilience, which are applicable along the physician career spectrum. Additionally, the open discussion nature of this curriculum helps normalize the discussion of these topics as well as showing that we all struggle at times in our careers. Dyrbye reports that students were more likely to seek help if they saw others reveal their own personal struggles [5]. Although not studied in this pilot, this witnessing of the struggles of peers could help reduce the stigma of admitting to PBO and improve the rates of trainees with PBO seeking help if needed. Furthermore, the concept is not unique to *Scrubs* and can easily expanded to use of other media and is worth further evaluation.

This program was initially designed for an internal medicine residency program but would be applicable for any residency or fellowship program. Ideally, it would be conducted with trainees in the same program to take advantage of their personal relationships and trust with each other. However, we would recommend a mixed group of trainees from each year of training within that program to help provide different viewpoints and anecdotes. It can be conducted in groups of any size but 15–25 learners are an ideal size to allow for adequate expression of a variety of viewpoints while allowing each trainee to participate fully.

## Conclusions

We conclude that this novel longitudinal curriculum, based on facilitated discussion of a comedic medical drama achieved its goals. It was very easy to implement, was successful in sparking difficult conversations about PBO and showed a trend towards decreased PBO among the trainees. The true value of this program was in facilitating trainee-led discussion of their own experiences and challenges, creating a sense of community that will hopefully help reduce the stigma of PBO. This pilot provides the framework for a comprehensive curriculum and this novel approach deserves further study. Given the overall success, our plan is to expand this particular curriculum to training programs at other hospitals, including multi-disciplinary sessions to address resident/nurse communication, an ICU specific version for doctors and nurses, and a faculty development version.

## Supplementary Information

**Additional file 1.** Faculty episode guide.

## Data Availability

Data sharing is not applicable to this article as no datasets were generated or analyzed during the current study.

## Abbreviations

PBO: Physician Burnout
ACGME: Accreditation Council on Graduate Medical Education
