# Supplementary material for: I’m No Superman: fostering physician resilience through guided group discussion of Scrubs
Source: BMC Med Educ. 2021 Aug 6;21:419. doi: 10.1186/s12909-021-02856-9 (PMC8344157; doi:10.1186/s12909-021-02856-9)
Supplement: Supplementary file 1 — Additional file 1. Faculty episode guide. [file 12909_2021_2856_MOESM1_ESM.docx]

**I'm No Superman: Fostering Physician Resilience through Guided Group Discussion of *Scrubs***

Arthur Holtzclaw, MD, Jack Ellis, MD, Christopher Colombo, MD

Appendix A: Faculty Episode Guide (Online Supplemental Material)

**Introduction:**

This document contains the pre-made faculty guide to use while facilitating the sessions. Each guide is two pages. The first page contains a summary of the episode and intro questions. The second page has a table with pre-set stopping points with open-ended facilitating questions for the faculty facilitator to ask to spark conversation as well as an area to record useful questions/comments that arise during the discussion. Several episodes have two separate guides, focusing on different parts of the episode.

**Tips and Pearls for success:**

1. Be cognizant of group size: 12-24 is usually optimal.
2. Be cognizant of group makeup: a range of PGY levels works best.
3. Protected time is essential as it allowed the residents to relax and prevented frequent disruptions of the discussion.
4. If done at lunchtime, consider including a low-cost lunch to increase attendance and facilitate a relaxed environment
5. Resist the urge to talk: It’s not a lecture, but a facilitated discussion. Uncomfortable silence is truly golden.
6. Review the episode ahead of time. Ensure you have the ability to see the time stamp on the episode to ensure accurate starts/stops.
7. Begin each session with ground-rules:
   1. All participants should be re-assured that the discussion will be a confidential forum.
   2. All participants should be reminded to share honestly, but to be respectful of others.
8. Give permission to your attendees to respond honestly: some of these topics are emotionally and spiritually provocative. Psychological safety of the participants is paramount to making this activity a resilience promoting and not a resilience degrading activity.
9. Feel free to not use every stop point! For most of the episodes there are more stops and discussion points than feasible in a 45-60 time slot. We suggested multiple stops and questions to allow the facilitator many options for stopping and allowing the discussion to develop organically according to the needs of the participants.
10. Most of all, be flexible. Sometimes the group will focus on an entirely different theme than was initially planned. If it is generating a good discussion, allow the housestaff to continue as it can be more pertinent to their current concerns/difficulties than the pre-planned topic. However, be ready to step in and guide the conversation as needed to avoid conflict between residents and/or ineffective discussion.

**Table of Contents and themes:**

1. S1E1: My First Day
   1. Key themes: First Day, Medical mistakes, collaboration vs competition, toxic leaders
2. S1E2: My Mentor
   1. Key Themes: Doctor/Nurse conflict, internship, mentorship
3. S1E4: My Old Lady
   1. Key Themes: Confidence, Death, Dealing with fear
4. S1E8: My Fifteen Minutes (full episode guide)
   1. Key Themes: Racism, community, sense of belonging
5. S1E8: My Fifteen Minutes (focused episode guide)
   1. Key Themes: Self-evaluation, dealing with criticism
6. S1E10: My Nickname (workplace dynamics focus)
   1. Key Themes: Workplace dynamics, annoying patient, critical self-evaluation
7. S1E10: My Nickname (dealing with the annoying patient focus)
   1. Key Themes: annoying patient, critical self-evaluation
8. S1E11: My Own Personal Jesus (full episode)
   1. Key Themes: Holidays, Faith, Sexism
9. S1E11: My Own Personal Jesus (focused episode)
   1. Key Themes: Sexism in medicine
10. S1E12: My Blind Date
    1. Key Themes: Confidence, disagreeing with an attending, taking risks
11. S2E5: My New Coat
    1. Key Themes: Identity, confidence, toxic work environment
12. S2E22: My Dream Job
    1. Key Themes: Burnout, Perseverance, Sacrifice
13. S3E5: My Brother, Where Art Thou?
    1. Key Themes: Gallows humor, coping mechanisms

**Episode Title:** My First Day

**Season:** 1

**Episode number:** 1

**Main Themes:** First Day, Medical mistakes, collaboration vs competition, toxic leaders

**Episode Synopsis**

The series begins with the first day of internship at Sacred Heart Hospital. Our Protagonist and narrator John Dorian (JD for short), is beginning internal medicine. His best friend, Chris Turk is beginning General Surgery. JD and Turk meet Elliot Reed, a female intern beginning internal medicine. This first day includes meeting their resident, meeting the chief of medicine, and their wisecracking attending Dr. Cox. JD, Turk and Elliot deal with their transition in different ways, and multiple plot tensions are a result. While JD and Elliot hide in a closet during their first “code blue”, Turk tries “learn by doing”, and faking confidence…which hilariously results in defibrillating a patient who was asleep, and not actually dead. JD feels alone as Turk initially does not want to continue being roommates (as they have been in undergrad and medical school). JD and Elliot are at cross purposes (he collaborating, she competing) during rounds with the initially kindly chief of medicine. JD has a hilarious daydream interrupted by his attending yelling at him about his inability to dose regular strength Tylenol, and then calls JD out for having the nurses do all his work for him. JD has his first call night which includes the death of a patient, and he learns afterwards that the “kindly” chief of medicine is quite unkindly. These tensions are resolved, as Elliot apologizes and helps JD out of his conflict with the chief of medicine, Turk moves in with JD, and JD realizes that his initially malignant attending (Dr. Cox) is actually a very gruff mentor. He wraps up with the thought that he cannot get through residency alone.

**Intro questions to focus group on particular themes:**

This episode works well during intern orientation, to get the interns to admit out loud that there is far more to learn than what they already know. If there is a mixed audience (i.e. residents and upper levels with the interns) this episode works well to let them know that what they are feeling is normal. If it is just an intern group, it still works to help them realize that they are not alone in feeling overwhelmed by the mismatch in responsibility of a doctor with their lack of experience.

How confident do you feel on your first day in internship?

How concerned are you that you’ll make a mistake?

Are you nervous about calling your resident/attending with “dumb questions”?

How do you recognize a good mentor, teacher or leader?

Is Residency a team sport, or an individual endeavor?

**Time stops and scene descriptions to facilitate discussion:**

| **Scene image** | **DVD time** | **Question for group** | **Further direction to spark discussion** | **Group initiated comments** | **Group initiated questions** |
| --- | --- | --- | --- | --- | --- |
| I don’t know Jack | 00:48 | How did you feel on your first day? | Does med school adequately prepare you for the internship? |  |  |
| I’m a tool…  (you’re definitely not allowed to talk)  (On call pep talk) | 4:24  (5:33)  (14:54) | Have you worked for someone with a difficult personality? | Where is the line between hierarchy and patient safety? |  |  |
| Its regular strength Tylenol  (this is totally normal) | 7:23  (11:39) | When should you get help? Is there ever a dumb question that you shouldn’t page the attending with? | Where is the balance between appropriate and excessive supervision? |  |  |
| Deer in the headlights | 10:57 | Are you concerned about not knowing something in front of your resident? Attending? Patient? | Is residency collaborative or competitive? |  |  |
| First code | 12:23 | What do you do if you’re first at a code? |  |  |  |
| Carla responds to Elliot | 15:48 | What are your expectations for doctor/nurse interactions? | What are your expectations for issues of gender equality in the workplace? |  |  |
| Would you just pronounce him so I can go home? | 18:01 | How do you expect to cope with the first death of your patient? | Do you expect others to be jaded about death? |  |  |
| That’s your patient, doctor | 21:59 | Can mentors have a disconnect between their true intentions and your first impression? |  |  |  |
| Wrap and closing comments | 22:44 | Do you have a support system to get through training? | Are we closer to each other than other professions? |  |  |

**Episode Title:** My Mentor

**Season**: 1

**Episode number:** 2

**Main Themes:** Doctor/Nurse conflict, internship, mentorship

**Episode Synopsis**

The episode begins with a funny musical sequence during which JD starts to imagine all the tasks in the hospital are coordinated to the beat of a song he is listening to. He expresses that he’s starting to “figure out” internship as tasks that were scary are now becoming routine. Elliot struggles with her difficulty avoiding verbal faux pas in social and work situations (insulting Turk’s mother inadvertently), and managing professional relationships with the nursing staff, Carla in particular (Elliot “tells on her” to Dr. Kelso). JD and Dr. Cox are taking care of a young patient (Will) with pneumonia and a smoking history that places him at risk of lung cancer. Turk begins to woo Carla and is rebuffed repeatedly. Elliot’s conflict with Carla continues to escalate with the nurses deciding to target her (for instance with a series of phantom pages to multiple floors of the hospital). JD tries to get through to Will to get him to quit smoking, and Will seems to accept the counsel initially. JD seeks validation from Cox for his success, and they both catch Will smoking in the stairwell. JD perseverates on “fixing Will” and interrupts Cox repeatedly for help. Elliot apologizes to Carla for telling on her to the Chief of Medicine, but then makes the situation worse by not leaving well enough alone. She even goes to the point of giving Carla credit for a good catch that Kelso falsely presumed was Elliot’s good catch. Turk impresses Carla by standing up to her on Elliot’s behalf, and encourages Carla and Elliot to come to a peace of sorts. After JD’s perseverance culminates in showing up at Dr. Cox’s home, Cox counsels JD that he needs to pick his battles by focusing on things he can change but then later frustrates him by giving him conflicting advice encouraging him to follow his convictions. The episode ends with a daydream of JD’s head actually exploding.

**Intro questions to focus group on particular themes:**

This episode works well during July/august time frame, once the Interns start to get into a routine. If there is a mixed audience (i.e. residents and upper levels with the interns) this episode works well to let them know that feeling repeated waves of routine punctuated by new frustrations is normal. If it is just an intern group, it still works to help them realize that they are not alone in feeling overwhelmed by the trap of needing to “save everyone”, or never quite feeling like they’ve “got it”. It is also about this point in training that recommending choosing/exploring a mentor will resonate, as Interns will realize how much they’re in for only after being in the hospital/clinic for a few months. This episode has multiple themes and not all stop points need to be used.

Do you feel like you’re developing a routine? What new frustrations have you found?

Are you going to be able to save everyone?

How do you recognize a good mentor, teacher or leader?

How do you handle inevitable conflicts/miscommunications with other professionals?

**Time stops and scene descriptions to facilitate discussion:**

| **Scene image** | **DVD time** | **Question for group** | **Further direction to spark discussion** | **Group initiated comments** | **Group initiated questions** |
| --- | --- | --- | --- | --- | --- |
| When you say the word Cancer, everyone reacts the same way…like that | 5:33 | Have you had to give anyone bad news yet? | Does a connection with a patient help or hurt your ability to care for them? |  |  |
| Sugar won’t work cuz they’re already so sweet  (Elliot digging grave)  (Nurses laughing during barrage of pages) | 7:08  (8:50)  (10:09) | How do you manage multidisciplinary care?  Do you manage conflict by pursuing peace, or victory? | Is the doctor the “Boss”?  Can use any or all of these stop times. |  |  |
| I thought it was a nice moment | 8:10 | What are your expectations for a mentor? |  |  |  |
| Private Party | 11:26 | Do you expect your patients to be truthful with you? | How does it make you feel when they aren’t? |  |  |
| GO! (Dr. Cox kicks JD out) | 12:40 | How do you approach a mentor for help? |  |  |  |
| Do you want to be the little spoon or big spoon? | 17:50 | Where do you set boundaries with a mentor? |  |  |  |
| Probably Stress | 21:05 | Does your passion help or hurt your longevity as a physician? | Do you engage with family/strangers as if they were patients? |  |  |
| End credits |  | Does your mentor ever drive you crazy? |  |  |  |

**Episode Title:** My Old Lady

**Season:** 1

**Episode number:** 4

**Main Themes**: Confidence, Death, Dealing with fear

**Episode Synopsis**

This episode begins with a lighthearted scene about intervening in a conflict that one should avoid. The true theme of the episode begins following the opening credits with a narration that one in three admitted patients will die and JD, Elliot and Turk each get a newly admitted patient. JD rapidly bonds with his patient, an elderly lady with renal failure who needs dialysis and he strongly encourages Turk do the same with his patient. Following his advice, Turk watches football with his patient, a young man with a hernia and quickly becomes friends with him. Meanwhile, Elliot struggles with her confidence in treating a Spanish speaking lady with lupus and a large PE.

After halfway into the episode, all three patients begin to have life-threatening issues. JD’s patient declines life-saving dialysis and he struggles with her decision to allow natural death to the point where she ends up comforting him. Turk’s patient is unexpectedly found to have lymphoma and Dr. Kelso yells at him for becoming friends with his patient. Elliot’s patient becomes hemodynamically unstable but Elliot’s fear paralyzes her to the point where she has difficulty even choosing which soda to drink, much less which treatment to give. Ultimately, all three patients die and the episode ends with a discussion of the importance of learning from each death.

**Intro questions to focus group on particular themes:**

This episode is one of the more powerful and is typically the highest regarded session by the residents. It covers the key themes of bonding with your patient, the fear as they decompensate and then coping with their death despite doing everything right. We typically introduce the episode by saying that this episode can provoke significant emotion and that it’s ok to express them. The key to facilitating this episode is to use silence and allow the residents to lead the conversation but open ended questions have been provided to spark conversation as necessary. Each time we have used this episode, we required more time than usual to decompress and it is important to make yourself and others available after the session to speak with residents if they desire.

How close do you get to patients and their families? Are they any concerns about becoming “too close”?

How do you deal with patients who deny lifesaving care that you think is appropriate?

How do you deal with the fear and indecision that arises while treating a decompensating patient?

How do you deal with death and what can you learn from it?

**Time stops and scene descriptions to facilitate discussion:**

| **Scene image** | **DVD time** | **Question for group** | **Further direction to spark discussion** | **Group initiated comments** | **Group initiated questions** |
| --- | --- | --- | --- | --- | --- |
| He must feel so safe and taken care of | 4:35 | How do connect with your patients? | What are the risks/benefits to balance when deciding how close to get to your patients, knowing they might die? |  |  |
| Ton of bricks for Dr. Dorian | 11:59 | Has a patient ever said anything to you like this? | Are you more concerned about a patient who is refuses treatment or a patient who says “do everything”? |  |  |
| I think I’m in trouble here… | 17:21 | Have you ever felt paralyzed by fear? | Are you more concerned about harming a patient through errors of omission or commission? |  |  |
| Hallelujah | 20:18 | How do you tell a family that their loved one has died? | Is it ok to display emotion and be comforted by the family? |  |  |
| What do you take away? | 21:15 | How do you cope with the death of a patient? | What have you taken away from the death of a patient? |  |  |

**Episode Title**: My Fifteen Minutes (Full episode)

**Season:** 1

**Episode number**: 8

**Key Themes**: Racism, community, sense of belonging

**Episode Synopsis:**

This episode begins with JD and Turk headed out for a lunch at a strip club, and ending up on the news initially commenting on a protest, and then resuscitating the cameraman. Dr. Kelso takes the opportunity of two of his interns gaining notoriety to start a marketing campaign to benefit the hospital. What starts out as a seemingly harmless and innocuous idea is actually a ploy to exploit Dr. Turk primarily as an African American and less so as a talented and intelligent surgery resident. Throughout the episode the interns are discussing their “intern evaluations”. JD has asked Dr. Cox to perform his, and is initially told to “do it himself” as Cox is too busy. Elliott has an awkward interaction with Carla in which she invites herself to a dinner at which she is not wanted. Carla fakes an excuse to get out of the dinner meeting, and then Elliott runs into Carla and her friend and realizes she was lied to in order to be excluded. The episode’s plot lines culminate with Dr. Turk convincing Dr. Kelso to drop the marketing campaign (in a rather funny scene involving Ted the hospital Lawyer), Elliot ends up treating Carla’s friend and becoming closer to Carla, and realizing that the nursing staff and physicians at Sacred Heart accept her as one of their own. JD eventually gets evaluated by Dr. Cox in a quintessential mentor scene in which Dr. Cox extols the virtues of pointed and honest self-assessment, as this is the real skill that a lifelong learner and professional must have in order to fulfill their potential.

**Intro questions to focus group on particular themes:**

This episode works well any time during the year. This guide is set up to focus on cultural sensitivity/racial tension scenes, with stop times provided for allowing a guided discussion on racial tension, and belonging-themed scenes. There is also a subsequent guide focused on the self-evaluation theme to use as desired as this theme is much easier to do in a focused session. This episode can be very emotionally charged to view/discuss. It is recommended to have additional time set aside to prevent any perception by the residents that short shrift are being given to these issues. Additionally, it is helpful to introduce the episode by reinforcing that the discussion should be honest, but that dignity and respect should be the foremost goal in expressing any comments. This episode is potentially double edged, in that it can diffuse tension regarding an uncomfortable topic with humor, but some may be offended that the issues are treated with humor at all. The key to this episode is emphasizing that a feeling of belonging is crucial to surviving in medicine, and that making people feel like “other” degrades their well-being and effectiveness as a clinician.

Do you feel that there are significant issues of cultural/racial/gender sensitivity in medicine?

Do you have any experiences in which stereotypes affected your practice of medicine or functioning at the workplace?

Have you gone out of your way to make someone feel like they belong, or to make someone feel like an outsider?

**Time stops and scene descriptions to facilitate discussion:**

| **Scene image** | **DVD time** | **Question for group** | **Further direction to spark discussion** | **Group initiated comments** | **Group initiated questions** |
| --- | --- | --- | --- | --- | --- |
| She’s not gonna get the invite…just hold your breath until it’s over | 3:14 | Have you been part of an awkward social situation at work? | Why might Carla not want to Invite Elliot? |  |  |
| Oh…I was Close | 5:56 | What cultural/racial/class stereotypes have you encountered in the workplace? | Do you consider the intent behind people’s cultural faux pas? |  |  |
| And I love ZZ Top | 7:30 | Do people express themselves differently when they think nobody is listening? | Are thoughts or actions more important in ensuring cultural/racial/gender sensitivity? |  |  |
| Turk sees the Wellness thru diversity | 10:07 | Use an uncomfortable silence, and an open ended “any thoughts?” |  |  |  |
| We both do… | 10:38 | Where is the line between culture and stereotypes? | Uncomfortable silence and open ended “any thoughts?” |  |  |
| You’re such a guy… | 13:20 | Does Carla have any prejudices regarding men? | Can a member of a minority exhibit insensitivity? |  |  |
| Dr. Turk will be driving your Beemer home to his place… | 16:09 | Is this an appropriate way to advocate for yourself? | How in your institution do you address issues of cultural/racial/gender insensitivity? |  |  |
| It’s not like I’ve ever fit in anywhere… | 17:13 | Do you feel like you belong here? | How important is a feeling of belonging in medicine? |  |  |

**Episode Title:** My Fifteen Minutes (Focused episode)

**Season:** 1

**Episode number**: 8

**Key Themes**: Self-evaluation, dealing with criticism

**Episode Synopsis:**

This episode begins with JD and Turk headed out for a lunch at a strip club, and ending up on the news initially commenting on a protest, and then resuscitating the cameraman. Dr. Kelso takes the opportunity of two of his interns gaining notoriety to start a marketing campaign to benefit the hospital. What starts out as a seemingly harmless and innocuous idea is actually a ploy to exploit Dr. Turk primarily as an African American and less so as a talented and intelligent surgery resident. Throughout the episode the interns are discussing their “intern evaluations”. JD has asked Dr. Cox to perform his, and is initially told to “do it himself” as Cox is too busy. Elliott has an awkward interaction with Carla in which she invites herself to a dinner at which she is not wanted. Carla fakes an excuse to get out of the dinner meeting, and then Elliott runs into Carla and her friend and realizes she was lied to in order to be excluded. The episode’s plot lines culminate with Dr. Turk convincing Dr. Kelso to drop the marketing campaign (in a rather funny scene involving Ted the hospital Lawyer), Elliot ends up treating Carla’s friend and becoming closer to Carla, and realizing that the nursing staff and physicians at Sacred Heart accept her as one of their own. JD eventually gets evaluated by Dr. Cox in a quintessential mentor scene in which Dr. Cox extols the virtues of pointed and honest self-assessment, as this is the real skill that a lifelong learner and professional must have in order to fulfill their potential.

**Intro questions to focus group on particular themes:**

This episode works well during the latter half of intern year, to get the interns ready for the increased self-scrutiny and self-assessment needed to transition to being a resident. It is also the time of year where end of year evaluations are approaching and the residents will have to evaluate others for perhaps the first time. The difficulty with this episode is that the themes tend to be seen as very disparate and unrelated. This guide is set up to focus on the self-assessment scenes, and a start and stop time is provided for the evaluation-themed scenes, so to maximize focus on evaluations, skip the intervening segments. A full episode guide is also available, allowing a guided discussion on the racial tension, and belonging-themed scenes. It is probably optimal to do the full episode first, to avoid the idea that the faculty are glossing over racial/gender/identity tensions to focus on evaluations, and then follow later in the year with the focus on self-evaluation.

How do you preferred to be evaluated?

Are you tense and nervous about evaluation?

Are you too easy or too hard on yourself during self-assessment?

**Time stops and scene descriptions to facilitate discussion:**

| **Scene image** | **DVD time** | **Question for group** | **Further direction to spark discussion** | **Group initiated comments** | **Group initiated questions** |
| --- | --- | --- | --- | --- | --- |
| Life in a hospital moves so …obsessing about intern evaluations (start) Opening Credits (end). | 1:21-2:12 | How do you feel about getting formal evaluations? | Do you feel like it’s a “grade” like in college or med school? |  |  |
| Noobie...COME! (start) Just ask the nurses for help (end) | 3:42-4:16 | Do you think it’s fair to ask an intern to self-assess? |  |  |  |
| I can’t evaluate myself (start)…CUZ I’M THE INTERN (end) | 6:00-7:08 | Are you too easy or too hard on yourself during self-assessment? | Why do you think Dr. Cox is being so passive aggressive? |  |  |
| When I saw it was missing?(start)  JD barks and takes back his evaluation (end) | 8:18-9:16 | What are barriers to self-evaluation? | What are barriers to evaluation in general? |  |  |
| And that’s when I realized (start)Buddy boy  Oh Danger. Oh (end) | 11:58-12:40 | Appropriate way to stand up for yourself? |  |  |  |
| Stay calm (start)End Credits (end) | 17:27-19:04 | What is the point of evaluation? | What is the difference between intrinsic vs extrinsic motivation? |  |  |
| Of course I knew exactly…(start) end credits (end)_ | 20:13-end | Can appearances be deceiving with Mentors? |  |  |  |

**Episode Title**: My Nickname

**Season**: 1

**Episode number:** 10

**Main Themes**: Workplace dynamics, annoying patient, critical self-evaluation

**Episode Synopsis**

This episode has multiple underlying sub-themes: workplace dynamics and friendships; dealing with the annoying patient; seeing ourselves through the eyes of others; learning to give ourselves a break. However, the overarching theme is critical self-evaluation. JD realizes that his medical knowledge has surpassed Carla's and Carla realizes she feels threatened by this, leading to conflict in their relationship. Meanwhile, Turk is forced to stand up to Dr. Kelso about eating his lunch on a public bench. A separate plot line involves the introduction of Jill, an annoying patient that is taken care of by Elliot and Dr. Cox throughout the series. Jill has multiple medical issues that arise from her stress, anxiety and inability to take a break. After Elliott identifies with her, she allows Jill to stay for the weekend. Dr. Cox challenges Elliot to justify that decision and helps her realize that she also needs to take a break from her self-criticism. Ultimately each character is forced to re-evaluate themselves through someone else’s eyes and gains a new understanding of each other.

**Intro questions to focus group on particular themes:**

This episode can take many paths and can be used to address the individual sub-themes or the main theme as a whole. Two episode guides have been written: one addressing work place dynamics and the other addressing dealing with an annoying patient. Of course, they can be combined into one session as well, based on the desired focus of the conversation. This guide addresses the work place dynamics.

Do you think you're better than nurses or sometimes behave as if you are?

Do you forget that they have a different perspective/background/knowledge base?

How do you deal with conflict with people at work? Does it change if it’s a high-level attending?

**Time stops and scene descriptions to facilitate discussion:**

| **Scene image** | **DVD time** | **Question for group** | **Further direction to spark discussion** | **Group initiated comments** | **Group initiated questions** |
| --- | --- | --- | --- | --- | --- |
| Everyone stops after JD corrects Carla: “He only needs Lasix”  OR  “They grow up so fast” and JD picks up Kleenex from floor | 2:44  OR  3:05 | Have you had this experience? | Have you or do you have friendship with nurses/PAs/NPs/PT/RTs?  Have you noticed a shift in the dynamics in these friendships as your training progresses? |  |  |
| Dr. Kelso on a bench with Turk  “Simpler times” | 7:21 | Have you ever had an implied request like this from someone higher in the hierarchy? | How do you deal with conflict with a higher ranked individual? |  |  |
| JD to Turk: Go outside and stand up for yourself... | 10:14 | Is it okay to stand up for someone above you in the hierarchy? Below you? |  |  |  |
| JD to Carla: “I’ll be the doctor and you be the nurse…. “yes Doctor” | 13:48 | How do you establish roles and respect in situations where you are friends as well as boss/subordinate? | How often do you let your emotions get the better of you? |  |  |
| Carla gets onto the bus after talking to JD  And JD stands in the rain | 20:44 | Silence: Group will usually initiate conversation after this | How much of your identity/self-worth is wrapped up in being a doctor?  Do you think you’re better than non-physicians? |  |  |
| End |  | Thoughts? | How do you navigate relationships with non-physicians at work? |  |  |

**Episode Title**: My Nickname (annoying patient focus)

**Season:** 1

**Episode number:** 10

**Main Themes:** annoying patient, critical self-evaluation

**Episode Synopsis**

This episode has multiple underlying sub-themes: workplace dynamics and friendships; dealing with the annoying patient; seeing ourselves through the eyes of others; learning to give ourselves a break. However, the overarching theme is critical self-evaluation. JD realizes that his medical knowledge has surpassed Carla's and Carla realizes she feels threatened by this, leading to conflict in their relationship. Meanwhile, Turk is forced to stand up to Dr. Kelso about eating his lunch on a public bench. A separate plot line involves the introduction of Jill, an annoying patient that is taken care of by Elliot and Dr. Cox throughout the series. Jill has multiple medical issues that arise from her stress, anxiety and inability to take a break. After Elliott identifies with her, she allows Jill to stay for the weekend. Dr. Cox forces Elliot to justify that decision and helps her realize that she also needs to take a break from her self-criticism. Ultimately each character is forced to re-evaluate themselves through someone else’s eyes and gains a new understanding of each other.

**Intro questions to focus group on particular themes:**

This episode can take many paths and can be used to address the individual sub-themes or the main theme as a whole. Two episode guides have been written: one addressing work place dynamics and the other addressing dealing with an annoying patient. Of course, they can be combined into one session as well, based on the desired focus of the conversation. This guide addresses the annoying patient.

We’ve all had that one patient that drives us crazy. How do you deal with them?

How much does dealing that patient affect your medical care of them or even your interactions with your colleagues?

**Time stops and scene descriptions to facilitate discussion:**

| **Scene image** | **DVD time** | **Question for group** | **Further direction to spark discussion** | **Group initiated comments** | **Group initiated questions** |
| --- | --- | --- | --- | --- | --- |
| Dr. Cox storms out of room while Elliot and Jill giggle over their phones | 04:21 | Look familiar?  OR  Have you ever had that patient that just drives you crazy? | Have you had an experience where you found a patient annoying and someone else didn't (or vice versa) ...thoughts? |  |  |
| Dr. Cox is using JD as a punching bag | 07:00 | Do annoying patients impact your interactions with others?  Have you ever taken out your irritation over an annoying patient on others? | Has it ever impacted your medical care? If you see 'that' patient on your outpatient list, does it ruin your day? |  |  |
| “Fun time is officially over.” | 08:50 | Have you ever been too blunt with a patient because they annoy you (ie. state that you have lost your patience with them)? |  |  |  |
| Elliot to Jill: “We’re gonna keep you here for the weekend to get some rest” | 13:10 | Have you ever had that moment with the annoying patient where the reasons for some of their behavior is brought to light? Do you ever fear you’ve missed that moment? | Have you ever been tempted to do something like Elliot did? Is it wrong? |  |  |
| Dr. Cox to Elliot: “It’s ok to give yourself a break once in a while” | 18:44 | Do you ever see yourself in your annoying patient? | Does identifying with your patients improve your attitude towards them? |  |  |

**Episode Title:** My Personal Jesus

**Season:** 1

**Episode number:** 11

**Key Themes:** Holidays, Faith, Sexism

**Episode Synopsis**

The episode begins with setting the timeframe as “the holidays” and begins with another conflict between JD and the Janitor centering around “ruining the holidays”. Elliot is working at the free clinic, and endures an inappropriate and offensive commentary from Dr. Kelso about women in medicine and the specialties that are female dominated. This becomes a robust plotline about Elliot struggling to reconcile her expectations, other’s expectations and a patient’s need, all in the context of gender discrimination by the chief of medicine (see focused episode guide for stop points to facilitate a discussion on issues of sexism). Turk begins the episode as excited about the holidays, as faith in god is quite central to his life, and his experience of the holidays. Following a particularly terrible call night, in which the suffering, injury and misfortune that befalls patients on a daily basis takes no pause for the holidays, Turk has a personal crisis in which he doubts his life-long beliefs. Carla attempts to help, but Turk becomes upset that she doesn’t understand how he truly feels. The episode culminates with Meredith (the pregnant patient) giving birth in the park after Turk is inspired to look for her there.

**Intro questions to focus group on particular themes:**

This can be an emotionally impactful episode for housestaff, particularly interns as this may be the first time in their life when they will have to work one or more of the major holidays and join the countless ranks of those whose holidays may be no different (or actually worse) than a regular workday. It can be especially helpful to have upper levels with the interns to pass on lessons learned in coping with holidays, and build a sense of community/family in your program. Of note, a focused episode guide is also available, focusing on the sexism that Dr. Kelso displays towards Elliot. It is probably optimal to either do the focused episode first or combine the two guides, to avoid the idea that the faculty are glossing over sexism and focusing on the holidays.

How do you cope with working during the holidays?

Does faith in something greater than yourself impact you as a doctor?

**Time stops and scene descriptions to facilitate discussion:**

| **Scene image** | **DVD time** | **Question for group** | **Further direction to spark discussion** | **Group initiated comments** | **Group initiated questions** |
| --- | --- | --- | --- | --- | --- |
| Elliot in the free clinic scene, Dr. Kelso’ sexist rant | 2:35 | Have you experienced sexist commentary in the workplace? | Consider leaving this stop out if you have already done the episode with the focused episode guide. |  |  |
| JD as Fonzy “ayyyyy” | 3:51 | Do families ever have these inappropriate expectations of medical providers? |  |  |  |
| Full love embargo baby! | 5:17 | Is faith important in your life? | How do you manage differences in beliefs? |  |  |
| All the spirit you need is right here | 7:35 | Do you feel the same about the holidays as you did while in college/medical school? | Is work harder during times where you “are supposed” to be happy or with family? |  |  |
| Turk post call | 13:15 | Allow silence | Do holidays affect your outlook? |  |  |
| I feel abandoned!....  Answer me please! | 17:20 | Do folks try to make you feel better without knowing what’s wrong first?  Do we ever do that with our patients or their families? | If you have faith in something, how do you manage seeming conflicts between your beliefs and the medical realities of our profession |  |  |
| God bless us, everyone | End credits | How does Turk’s reliance on faith make you feel? | What keeps you going when things get difficult? |  |  |

**Episode Title:** My Personal Jesus (focused episode)

**Season:** 1

**Episode number:** 11

**Key Themes:** Sexism

**Episode Synopsis**

The episode begins with setting the timeframe as “the holidays” and begins with another conflict between JD and the Janitor centering around “ruining the holidays”. Elliot is working at the free clinic, and endures an inappropriate and offensive commentary from Dr. Kelso about women in medicine and the specialties that are female dominated. This becomes a robust plotline about Elliot struggling to reconcile her expectations, other’s expectations and a patient’s need, all in the context of gender discrimination by the chief of medicine. This guide focuses on this plot at the exclusion of the holiday and faith themes. In the climactic scene with Kelso, Elliot expresses that her primary concern is her patient, regardless of the patient (or her own gender). The episode culminates with Meredith the pregnant patient giving birth in the park after Turk is inspired to look for her there, and Elliot discovers that she can actually devote her life to Internal Medicine, and also be inspired and awed by the new life a baby represents.

**Intro questions to focus group on particular themes:**

This can be an emotionally impactful episode for housestaff, particularly interns, as the sexism Elliot deals with is quite explicit and offensive from Dr. Kelso. Splitting this plot out separately allows for more time devoted to discussion, or possible smaller group discussions, with then reconvening into a larger group to share thoughts.

Have you experienced sexism/discrimination/inappropriate comments in the workplace?

How do you reconcile your own gender role expectations with those of your employer, society, family?

**Time stops and scene descriptions to facilitate discussion:**

| **Scene image** | **DVD time** | **Question for group** | **Further direction to spark discussion** | **Group initiated comments** | **Group initiated questions** |
| --- | --- | --- | --- | --- | --- |
| Elliot in the free clinic scene, Dr. Kelso’ sexist rant | 1:21-2:35 | Have you experienced sexist commentary in the workplace? | Are Women in medicine subject to assumptions and expectations to include specialty?? |  |  |
| Elliott explains to her patient that she’s pregnant, and then runs out on her | 5:39-6:12 | Is this because Elliot is clinically uncomfortable, or reacting to what Kelso said? |  |  |  |
| Meredith has HELLP syndrome | 10:42-11:10 | How do you think Elliott handled this? |  |  |  |
| Elliot can’t find Meredith | 13:48-14:20 | This stop is intended for story continuity for the sexism discussion, not a stopping point for discussion |  |  |  |
| I’m a doctor who’s interested in her patient | 15:15-15:58 | Is this perhaps the perfect response to Kelso? |  |  |  |
| Elliott “saves” JD, after learning of a 911 call about a pregnant woman | 18:39- end | Is it ok that Elliot discovers that she does love babies?  How does Turk’s reliance on faith make you feel? |  |  |  |

**Episode Title:** My Blind Date

**Season:** 1

**Episode number:** 12

**Key Themes:** Confidence, disagreeing with an attending, taking risks

**Episode Synopsis**

The episode begins with a contentious but funny exchange between JD and his nemesis, the Janitor. A hospital employee slips and falls, before the episode moves forward with Dr. Cox rallying the housestaff and nursing staff to a Knute Rockne-esque pep talk in which Dr. Cox issues the ultimatum: no ICU patient will die overnight because he is hoping for the perfect game. All 27 ICU patients are alive at the beginning of his shift, and he intends to have all 27 patients still alive at the end of the shift (27 is the number of batters successfully retired by the pitcher in a “perfect game” in baseball). Dr. Cox interacts in terse and challenging ways to motivate the housestaff to assist his quest for perfection, with varying responses (Doug decompensates, JD calmly manages his tasks, and Elliot becomes increasingly frustrated at her inability to “get in the game”.) JD is asked to tend to the patient that slipped and fell in the first scene by Dr. Kelso, creating an opportunity for Elliot to step up as Dr. Cox’s “Go-to intern”. Carla and Turk have a side plot in regards to some tension in their relationship that eventually resolves with Turk admitting to Carla that he has fallen in love with her.

The episode progresses with Elliot stepping into the role of Cox’s main assist for the remainder of his shift and culminates with a patient dying during an attempted resuscitation by Dr. Cox and Elliot, unfortunately just before midnight. Cox essentially admits that the pursuit of the “perfect game” is unattainable, but is the ideal for which we should strive. He begins to treat Elliot as the capable and hardworking physician we know her to be.

**Intro questions to focus group on particular themes:**

Does the death of a patient automatically equate to a failure?

Is the pursuit of perfection an unattainable set up for failure, or an inspiration in order to get the very best from ourselves/our teams?

How do you best mentor a protégé to develop confidence?

**Time stops and scene descriptions to facilitate discussion:**

| **Scene image** | **DVD time** | **Question for group** | **Further direction to spark discussion** | **Group initiated comments** | **Group initiated questions** |
| --- | --- | --- | --- | --- | --- |
| Death as an annoying coworker | 3:17 | How do you view death when taking care of truly sick people? | Is death a failure, or an opportunity? |  |  |
| I’m your wing man, Maverick | 3:54 | How did each resident react to Cox’s challenges? | Is he developing or denigrating? |  |  |
| Elliot as the “catcher” for Dr. Cox. | 11:55 | Do you assist your peers in gaining opportunities to learn/grow/shine? | Are high stakes situations the best time for this? |  |  |
| You know what they say about first instincts?...  Should I? | 14:03 | Do you think there was a purpose to Dr. Cox causing Elliot to second guess herself? | Is this an effective mentor tactic? |  |  |
| “Get back in the game Elliot” | 16:20 | What do you think about disagreeing w/ your attendings like JD suggests? | How do you build confidence in your juniors? |  |  |
| No, I won't call it. | 18:43 | What is more impactful: praise for an unauthentic triumph, or praise for a sincere effort in defeat? |  |  |  |
| New game starts in four minutes | 19:14 | (Allow silence) | How do you move on after a patient death? |  |  |
| End credits |  | What do you think of JD's comment about 'every time you take a big risk, it's worth it? | Was becoming a doctor a big risk? |  |  |

**Episode Title:** My New Coat

**Season**: 2

**Episode number:** 5

**Main Themes:** Identity, confidence, toxic work environment

**Episode Synopsis**

This episode deals with the struggles that new doctors have in establishing their identity within the hospital. The pre-opening credit scenes introduce the three main arcs in the episode: JD beginning to act more confident now that he is a resident but then dealing with a bad patient outcome, Elliot dealing with how people see her after she begins a relationship with another doctor, and Turk trying to fit in with the surgical “boy’s club” and making comments that aren’t in his character.

JD’s arc is the primary focus with him using his new confidence to stand up to the Janitor and then Dr. Cox who tries to mock JD in front of a patient for making a mistake. JD later gains the trust of that patient and convinces him to take imipenem but then the patient blames JD for his subsequent anosmia. When JD realizes it wasn’t his fault, he tries to gloat to Dr. Cox who tells him that sometimes being a doctor means allowing the patient to achieve some solace by blaming you. JD ends up taking the blame for it and healing his relationship with the patient.

Meanwhile, Turk is stuck working with the short surgeon due to missing guy’s night and he attempts to get back into the “boy’s club” by speaking poorly about Elliot who overhears him. The short surgeon explains to Turk that he can still be a good surgeon without being part of the boy’s club and he stands up to the other surgeons and defends Elliot. He also apologizes to Elliot for what he did.

Elliot sleeps with a staff surgeon and is called multiple names by the other staff. At first, she is angry but later is happy that she at least has an identity now. This lack of confidence and eagerness to be noticed is a frequent theme for Elliot throughout the series. The episode ends with the description of the different ways that people try to establish their identity and how it affects their actions.

**Intro questions to focus group on particular themes:**

This episode works well early in the academic year as interns and residents are growing into their roles as junior doctors and trying to establish themselves in the hospital. This episode is particularly useful with multiple levels of trainees as they can discuss the pressures unique to each year and challenges that they have overcome. JD’s arc is particularly pertinent to trainees as his difficulty with establishing himself as a doctor and taking ownership of his patient is fairly universal. His desire to show everyone that the poor outcome wasn’t his fault is also likely to spark significant discussion. Turk’s arc is particularly useful when discussing the importance of a non-toxic working environment and how easy it is for people to cause significant harm and distress to their coworkers with simple words. Elliot’s arc is exaggerated more than the others but could be used to show the impact of a lack of confidence on one’s overall psyche. We rhetorically introduced the following questions prior to the episode but started the episode without going into significant discussion of them.

How did you feel early in your first or second year as a doctor?

Did you struggle with your identity as a doctor?

What struggles have you encountered in fitting in at the hospital?

How have you felt when you think you’ve made a mistake?

**Time stops and scene descriptions to facilitate discussion:**

| **Scene image** | **DVD time** | **Question for group** | **Further direction to spark discussion** | **Group initiated comments** | **Group initiated questions** |
| --- | --- | --- | --- | --- | --- |
| “You see things have changed” | 1:07 | How important are the symbols of being a doctor?  Do people make you feel like a “fake” doctor? | Do you see any downsides to this sudden new confidence? |  |  |
| I would appreciate it if you wouldn’t stand here and yell at me in front of my patient…. | 4:40 | Has this ever happened to you?  Has anyone ever mocked you for making a mistake (even if it wasn’t your fault or you didn’t make one)?  How did you want to respond? | What is the best way to deal with a situation like this? |  |  |
| Turk talking about Elliot and getting high-fived | 10:04 | Have you ever been in a situation like this?  I. e. have you felt pressured into doing something that you knew was wrong in order to fit in? | What have been the consequences if you don’t give in? |  |  |
| No more silly mistakes. Damn | 12:12 | How many of you have experienced something like this? | How do you cope after you feel like you’ve made a mistake? |  |  |
| Doesn’t that seem like something that goes along with wearing that fancy coat | 17:38 | What do you think about this? | What is the role in allowing the patient to vent? |  |  |
| End credits |  | What difficulties have you had in establishing your identity at work? |  |  |  |

**Episode Title:** My Dream Job

**Season:** 2

**Episode number:** 22

**Key Themes:** Burnout, Perseverance, Sacrifice

**Episode Synopsis**

This episode takes placed during the final portion of their second year of residency and focuses on the monotony and difficulty of working in the medical field. It begins with Dr. Kelso berating Carla for being late and then points how much of residency is paperwork and prescribing the same three medications. This lack of excitement is accentuated by the arrival of one of JD and Turk’s college friends who takes them out drinking and ironically comments how they found their “dream jobs”. Dr. Reed accidently stabs Dr. Kelso in the face with a needle and is targeted by him incessantly, which breaks down her confidence. Meanwhile, the Janitor points out that he makes more money than JD. When their friend encourages them to go out drinking despite being on backup call, Turk and JD ultimately agree but are called in, leading to them being called out by Dr. Cox for their lack of professionalism. They initially try to blame their friend who points out that they just hate their job. They ultimately remember why they chose their careers and accept the sacrifices that go along with it. The episode ends with Dr. Cox punching Dr. Kelso in the face in an effort to show Elliot specifically and the residents generally that they are doing a good job.

There is a subplot in which Dr. Cox finds out that his ex-wife’s baby is actually his son. She had been telling him that it was another man’s baby so that he wouldn’t feel compelled to stay with her. This discovery helps him decide to continue to act as a father figure for the residents.

**Intro questions to focus group on particular themes:**

This episode works well during the second half of the academic year such as the winter when burnout rates are the highest or during the late spring as the academic year ends. The emphasis is on the difficulties we face in doing our jobs every day and the sacrifices that we are asked to make on a daily basis, leading to questioning on whether what we do is actually worth it. This emphasis resonated with our residents who expressed dealing with similar emotions throughout the year, particularly second year residents, who are most prone to feelings that medicine is drudgery. Interns will become residents, senior residents will become staff, and second years will have another year of being residents (per JD “it’s impossible to see the light at the end of the tunnel”.)

What were the reasons you first chose medicine as a career?

Are those the same reasons that you remain in medicine?

Do you feel that medicine is an honor, calling, vocation or a job?

Does it feel this way every day, or does it take reminding?

Does it feel different than when you had just started?

**Time stops and scene descriptions to facilitate discussion:**

| **Scene image** | **DVD time** | **Question for group** | **Further direction to spark discussion** | **Group initiated comments** | **Group initiated questions** |
| --- | --- | --- | --- | --- | --- |
| And of course the paperwork…..  OR  You guys pretty much landed your dream jobs didn’t you? | 1:53  4:54 | Is being a doctor everything you thought it would be? | Have you considered how life would be different in a different profession? |  |  |
| Dr. Reed, a moment please?  (Re-suture that wound…) | 6:48    (10:20) | Do you feel targeted by your attendings? | Where is the line between challenging and just being malignant? |  |  |
| Do you have any idea how to feels to have a janitor make more money than you? Johnny… investment banker, so… no | 8:38 | Do you feel underappreciated for the work that you do? | Is intrinsic or extrinsic motivation more important for medicine? |  |  |
| You’re not fit to work tonight | 12:55 | Why do you think they went out tonight? | How can feeling burnout affect our work? |  |  |
| You’re just pissed because you hate your jobs | 15:49 | How do you feel about being a doctor at this time? |  |  |  |
| I think you have this fathering thing down! | 19:46 | Do you appreciate “tough love” from mentors? | Is there a single optimal mentor style? |  |  |
| End credits |  | Who do you go to for help? | What are ways of coping with these common emotions? |  |  |

**Episode Title:** My Brother, Where Art Thou?

**Season:** 3

**Episode number**: 5

**Key Themes**: Gallows humor, coping mechanisms

**Episode Synopsis**

This episode begins with JD interviewing Mr. Bober, a 'frequent flyer" nursing home patient with dementia. Dr. Cox enters as JD is mocking the patient and tells JD that he's never been prouder. Dr. Cox then proceeds to complain about the frequent NH admissions, referring to the patients as "gomers". Fast forward, JD's brother joins him and Dr. Cox on rounds with the readmission of Mr. Bober. During rounds, JD’s brother asks what a gomer is to which JD replies "It's an old person who takes up room in the hospital and doesn't have the decency to die." His brother responds negatively to this, calling JD rude. JD then rants about that he doesn't have time for Mr. Pickles because he has 'three vegetables and a drug addict who's going to tell me this time things are going to be different." This later sets up a discussion between JD’s brother and Dr. Cox, emphasizing the importance of setting a good example for the trainees.

Meanwhile, the rest of the episode focuses on the everyday challenges that the other members face in addition to their typical responsibilities. Elliott and Carla are caught moonlighting while trying to earn enough money to see her boyfriend and for her wedding respectively and are facing unpaid suspension. Turk talks about the difficulty he has with family at the holidays. While not directly applicable to the main theme as above, it can be used as a reminder that life still goes on no matter what we are dealing with in the hospital.

**Intro questions to focus group on particular themes:**

This episode works well at any point in the year but especially in the second half when burnout rates are higher. The key theme is the use of gallows humor and how it can go too far and impact how we see our patients. It also addresses how it is seen very differently by the medical professionals using it (Dr. Cox and JD) versus the family members (JD’s brother) who is horrified by it.

What coping mechanisms do you use in your daily life at the hospital?

What coping mechanisms do you see used around you? Do you think some are more beneficial/maladaptive?

How do your coping mechanisms affect those working with you/under you?

Are there downsides to using humor as a coping mechanism?

**Time stops and scene descriptions to facilitate discussion:**

| **Scene image** | **DVD time** | **Question for group** | **Further direction to spark discussion** | **Group initiated comments** | **Group initiated questions** |
| --- | --- | --- | --- | --- | --- |
| I could just give you a hug...but I never would | 1:10 | What do you think about using gallows humor as a mode of survival? | How does the culture of medicine propagate its use? |  |  |
| Regarding the rum and coke issue. Couldn’t be more confused | 10:08 | Do you ever use gallows humor around non-medical friends of family?  How do you respond to non-medical individuals who question your humor? | At what point does the humor go too far/when do it cross the line? |  |  |
| I'm not sure I like the guy you're turning into... | 11: 55 | Has this job changed you? | For better or worse? |  |  |
| A month later we gonna do it again at Christmas. | 13:37 | Do you need support from non-medical family and friends or do they not “get it”? | Do they not get it, or do you not get it? |  |  |
| Get of my office before I change my mind | 16:40 | How does your life outside the hospital affect your actions/decisions? | Do you take the time to think about what other people have going on before judging their actions? |  |  |
| End credits | 19:51 | Allow silence (What do you think?) | What impact do you have on the people working with you? Under you? |  |  |
